# Flight toward Sustainability in Poultry Nutrition with Black Soldier Fly Larvae

**DOI:** 10.3390/ani14030510

**Published:** 2024-02-03

**Authors:** Md Salahuddin, Ahmed A. A. Abdel-Wareth, Kohzy Hiramatsu, Jeffery K. Tomberlin, Daylan Luza, Jayant Lohakare

**Affiliations:** 1 Poultry Center, Cooperative Agricultural Research Center, Prairie View A&M University, Prairie View, TX 77446, USA; mdsalahuddin@pvamu.edu (M.S.); dluza@pvamu.edu (D.L.); 2 Department of Animal and Poultry Production, Faculty of Agriculture, South Valley University, Qena 83523, Egypt; 3 Laboratory of Animal Functional Anatomy (LAFA), Faculty of Agriculture, Shinshu University, Kami-ina, Nagano 399-4598, Japan; seitaik@shinshu-u.ac.jp; 4 Center for Environmental Sustainability through Insect Farming, Texas A&M AgriLife, College Station, TX 77843, USA; jeffery.tomberlin@ag.tamu.edu

**Keywords:** broilers, black soldier fly larvae, sustainability, productivity

## Abstract

**Simple Summary:**

This review article comprehensively explores black soldier fly larvae (BSFL), *Hermetia illucens*, as a sustainable alternative protein source in poultry nutrition. It discusses the ecological distribution, nutritional composition, and benefits of BSFL, including their high digestibility and nutrient availability. This article highlights the impact of BSFL on broiler growth performance, meat quality, and gut health and underscores the importance of proper inclusion levels for optimal results. In conclusion, this study emphasizes that BSFL offer a nutritionally rich, environmentally sustainable solution, presenting a viable alternative to conventional feed sources and contributing significantly to sustainable agriculture and food security.

**Abstract:**

Black soldier fly larvae (BSFL), *Hermetia illucens* (L.) (Diptera: Stratiomyidae), have emerged as a promising feed ingredient in broiler chicken diets, known for their high protein content, nutritional richness, and environmental sustainability. This review examines the effects of integrating BSFL into broiler feeds, focusing on aspects such as growth performance, nutrient digestibility, physiological responses, and immune health. The ability of BSFL to transform waste into valuable biomass rich in proteins and lipids underscores their efficiency and ecological benefits. Protein levels in BSFL can range from 32% to 53%, varying with growth stage and diet, offering a robust source of amino acids essential for muscle development and growth in broilers. While the chitin in BSFL poses questions regarding digestibility, the overall impact on nutrient utilization is generally favorable. The inclusion of BSFL in diets has been shown to enhance growth rates, feed efficiency, and carcass quality in broilers, with the larvae’s balanced amino acid profile being particularly advantageous for muscle development. BSFL may also support gut health and immunity in broilers due to its bioactive components, potentially influencing the gut’s microbial composition and enhancing nutrient absorption and overall health. Moreover, the capacity of BSFL to efficiently convert organic waste into protein highlights their role as an environmentally sustainable protein source for broiler nutrition. Nonetheless, further research is necessary to fully understand the long-term effects of BSFL, ideal inclusion rates, and the impact of varying larval diets and rearing conditions. It is crucial for poultry producers to consult nutritionists and comply with local regulations when incorporating new feed ingredients like BSFL into poultry diets.

## 1. Introduction

By 2050, the world’s population is anticipated to reach 10 billion, leading to a rise in global food requirements by about 35% to 56% from 2010 levels, as reported by Van Dijk et al. [1]. Animal products currently account for nearly 70% of worldwide food consumption, a figure projected to grow, primarily due to changing dietary preferences and rising economic status, particularly in regions with lower incomes [2]. To accommodate this surge in demand for animal-based products, the global poultry industry needs to enhance its productivity and adopt more eco-friendly practices. Sustainable feeding practices are essential for the poultry sector to achieve environmental and economic sustainability. The choice and management of animal feed play a pivotal role in this process, influencing the overall impact of livestock production on the environment and its economic viability. The high cost of poultry feeds is primarily influenced by the scarcity of critical ingredients like soybean and fishmeal [3]. These components are favored in poultry diets due to their palatability and high protein content. However, their reliance on imports and the associated high costs have prompted researchers to explore alternative protein sources that could substitute soybean and fishmeal in poultry feeds.

In recent years, the quest for sustainable and environmentally friendly alternatives within the animal feed industry has led to a growing interest in BSFL, scientifically known as *Hermetia illucens* (L.) (Diptera: Stratiomyidae) [4,5,6,7]. This species, originating from South America, and its larvae have demonstrated remarkable adaptability across various climates, including temperate, subtropical, and tropical regions. Thriving best in temperatures of 25 °C to 30 °C, BSFL can also grow in a range of environmental conditions, showcasing their resilience. Their natural inclination toward habitats rich in decomposing organic material, coupled with this adaptability, highlights their pivotal role in sustainable agriculture and waste management, suitable for diverse geographical locations [8,9,10]. These larvae, which can grow up to 27 mm in length and weigh approximately 220 mg, are nutritionally dense, with a composition rich in proteins and fats [11,12,13,14,15,16,17]. This makes them an invaluable source of nutrients compared with conventional feed ingredients, suitable for a range of animals including poultry [18,19,20,21,22,23], aquaculture species such as fish [24,25,26,27], and swine [28,29,30]. Furthermore, BSFL significantly reduces waste volume by rapidly eating organic waste, providing a practical substitute for conventional landfill disposal techniques. This contributes to reducing methane emissions, a potent greenhouse gas frequently emitted by decomposing organic matter in landfills, and waste accumulation [31,32,33].

In the field of animal nutrition, BSFL have emerged as a highly viable and sustainable source of protein. They present an environmentally friendly alternative to conventional feed ingredients such as soybean meal and fishmeal, which are often linked to environmental challenges like deforestation and overfishing. Additionally, traditional feed components like soybean meal and fishmeal with other feed ingredients can account for up to 70% of total poultry production costs [34]. The nutrient-dense nature of BSFL, which is rich in proteins and lipids, makes them an excellent solution to the growing need for quality feed in chicken raising. The fact that BSFL can feed on various organic wastes not only demonstrates their sustainability but also contributes to significant waste reduction. The dual role of BSFL in delivering vital feed while reducing waste has piqued the interest of the animal feed industry. Their efficient waste conversion into usable biomass adheres to circular economy concepts, establishing BSFL as a trailblazer in advancing sustainable poultry farming operations. The nutritional benefits of BSFL have been shown to be the most appealing to the feed business, with a balanced amino acid composition and a high concentration of protein, energy, and mono- and poly-unsaturated fatty acids [35].

Therefore, this review gives an overview of practical insights on incorporating BSFL in broiler chicken diets, focusing on their nutritional content, processing techniques, and ideal inclusion levels. This article synthesizes findings from diverse research studies, providing an understanding of the impact of BSFL on poultry growth performance, meat quality, and gut health, specifically in broiler diets. It delves into the potential benefits and challenges associated with using BSFL. The conclusions and discussions presented are intended to inform future research and practical implementation of BSFL in poultry nutrition, thereby significantly contributing to the progression of sustainable livestock farming.

## 2. Overview of the Black Soldier Fly (BSF): Distribution, Biology, and Utilization

**Figure 1:**
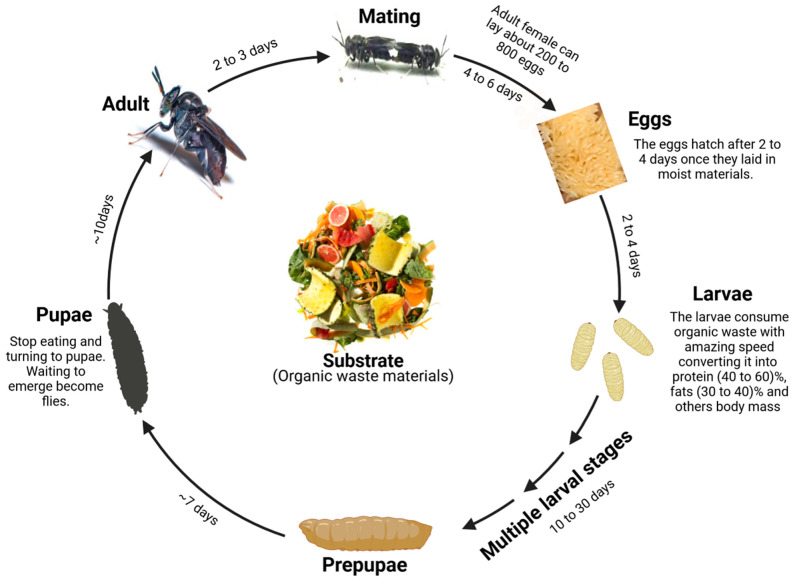
The life cycle of a black soldier fly (BSF). This diagram illustrates the complete metamorphosis stages of a BSF, starting from the egg stage, through multiple larval stages, pupation, and culminating in the adult form. The cycle begins with the adult female laying 200 to 800 eggs, which hatch after 2 to 4 days. The emerging larvae consume organic waste, rapidly growing and going through several molts before entering the pupal stage. After approximately 10 days, adult flies emerge from the pupae, ready to mate within 2–3 days, thus continuing the cycle.

The black soldier fly (BSF) is a distinct member of the Stratiomyidae family within the Diptera order. BSF, primarily thriving in South America, has adapted to a wide range of climates including temperate, subtropical, and tropical regions, with its ideal living conditions being temperatures between 25 °C and 30 °C [36,37]. Outside of industrial production, they cannot live in northwestern Europe and locations with temperatures below 5 °C due to their inability to withstand the cold [38]. Today, the BSF is estimated to inhabit over 80% of the world, particularly between latitudes 46 N and 42 S [39]. Incredibly prolific in humid tropical areas, the BSF is drawn to regions abundant in decomposing organic materials. As detailed by Seyedalmoosavi and colleagues [40], the life cycle of the BSF is holometabolous, encompassing four distinct stages: the egg, larva, pupa, and adult (Figure 1). These eggs are deposited in batches, usually ranging from 200 to 800, on moist organic-rich substrates. They hatch in two to four days, unleashing larvae with an insatiable taste for various organic leftovers such as food scraps, animal excrement, and slaughterhouse remains. These larvae are nutritionally rich, reaching sizes of up to 27 mm and weights approaching 220 mg [11,12,13,14,15,16,17]. Their growth is segmented into six instars, culminating in maturity between 10 to 30 days, a timeline influenced by ambient temperature and the quality of their substrate source [40]. Once matured, they cease feeding and seek out desolate, shadowed spots to pupate. Pupae are distinguishable by their brown hue and ovular shape, with typical lengths around 12 mm. This stage spans between 10 to 20 days, after which the adult flies emerge. These newly formed adults engage in mating rituals within days. Characterized by their ability to be maintained in a colony without providing adult nutrition and still reproduce, their lifespan is brief, lasting only about five to eight days [11] depending on the population and associated conditions. Aesthetically, these adults, with lengths ranging from 13 to 20 mm, exhibit a dark hue akin to wasps. Other features include elongated antennae and a notable translucent section on their abdominal region that results in the wasp-like appearance. Importantly, they pose no threat as pests and are not known to serve as vectors for pathogens. BSFL have been recognized as an innovative solution to several contemporary environmental and agricultural concerns, with their multifaceted applications documented extensively in the recent scientific literature [31,32]. One of their most noteworthy utilities lies in organic waste management. BSFL exhibit an impressive capacity to decompose a wide variety of organic materials. According to Diener et al. [41], these larvae can efficiently process food waste, agricultural residues, and animal manure. This rapid consumption significantly curtails the volume of organic waste, offering a sustainable alternative to traditional landfill disposal methods. In turn, this helps mitigate methane emissions, a potent greenhouse gas commonly released from decomposing organic matter in landfills [42]. Furthermore, as BSFL process this waste, they convert it into valuable biomass, rich in proteins and lipids. Several studies have shown that BSFL have the potential to be a high-quality protein source for poultry, fish, and swine [11,12,13,14,15,16,17,18,19,20,21,22,23,24,25,26,27,28,29,30]. Given their nutrient-dense content, as well as the sustainability of their production, they are an appealing alternative to conventional feed ingredients such as soybean meal and fishmeal. Furthermore, the lipid content of BSFL has been investigated for biodiesel generation. The fatty acid composition of BSFL-derived oil, according to Li et al. [43], is ideal for biodiesel synthesis, potentially delivering a renewable energy source from waste-derived biomass.

## 3. Biochemical Profile of BSF Larvae

The BSFL have been identified as a potential source of both protein and fats, offering considerable benefits for use in livestock feeds. The specific composition of these larvae can be attributed to a range of factors including the type and quality of the feed they consume, their growth phase, and the environmental conditions they are exposed to. Generally, the larvae’s nutritional composition is characterized by a protein content ranging from 32% to 53% and fat levels ranging from 18% to 33% [13,44,45]. The notable fat content of BSFL significantly influences poultry feed formulation, necessitating adjustments in nutrient balance and energy density [46]. This high level of fat can also impact pellet processing, affecting aspects such as pellet stability and nutrient preservation [47]. Consequently, incorporating BSFL into poultry diets requires careful consideration of these factors. Feed formulators need to account for the higher energy content associated with BSFL and the potential effects on pellet quality [11]. Ensuring nutritional adequacy while leveraging the benefits offered by BSFL fat content is crucial [14]. These larvae are particularly notable for their rich content of essential amino acids and a high concentration of lauric acid. Additionally, they can absorb a range of micronutrients from their diet, such as different minerals and vitamins [48,49]. Developing practical guidelines for integrating BSFL into poultry diets, considering their unique nutritional profile, is essential to achieve optimal feed production and support poultry health.

BSFL are used in poultry feed in several forms, each tailored to meet specific dietary needs and feeding strategies. The two main forms are whole insects and processed meals. Whole BSFL, whether dried or fresh, are a great source of protein and fat, and they encourage natural foraging behavior in poultry. Processed BSFL meal, which is finely ground, offers a consistent delivery of nutrients, ideal for uniform feed formulations. Within these forms, BSFL can be found as either defatted or full-fat. Defatted BSFL have less fat, making them a good choice for diets requiring lower fat levels. Full-fat BSFL retain their natural fat content, including saturated fats, offering a richer energy source. These options allow for flexibility in feed formulation, enabling producers to choose the best form of BSFL based on the specific nutritional requirements and goals of their poultry diets. The approximate chemical composition of BSFL is shown in Table 1. Studies indicate significant variability in the protein content of BSFL, as shown by Yildirim-Aksoy et al. [50] and Schiavone et al. [51], which report protein percentages ranging from a minimum of 21.6% to a maximum of 65.5%. Considering these levels, the protein content in BSFL can vary and is sometimes less than that found in standard soybean meal (49.4%) and fish meal (67.5%), as reported [52]. The full-fat larvae tend to have a more uniform protein content, while the defatted larvae display a wider range, potentially due to the impact of the defatting process on the protein structure. Looking at the fat content, full-fat BSF larvae show a spectrum from 29.4% as noted by Onsongo et al. [53], to 51.5% as indicated by Tyshko et al. [54], with an average of around 35.3%, which is considerably higher than that of conventional soybean meal at 1.4% and fish meal at 10.4% [52]. Even the defatted BSFL, with an average fat content of 6.9%, surpass the fat levels in standard soybean meal. Other nutritional elements such as ash are present on average at 8.2% in BSFL, exceeding the ash content in soybean meal (7.19%) but not as high as in fish meal (17.15%), according to the National Research Council [52]. The fiber content in BSFL averages 9.5%, ranging from 4.1% in studies by Spranghers et al. [55] to 21.3% as found by Onsongo et al. [53]. This fiber percentage is lower than soybean meal (7.43%) but higher than fish meal (0.26%). Chitin, an essential component of BSFL, averages 6.17% and varies from 3.87% to 7.21% as reported by Tyshko et al. [54]. This ingredient, which is mostly composed of N-acetyl-D-glucosamine units and has a molecular structure similar to cellulose, is typically indigestible but has been shown to strengthen animal immunity. The range of fiber content in BSFL is frequently linked to their developmental stage, with a propensity for increased fiber levels as they get closer to the adult stage of metamorphosis. As a result, various research investigations on BSFL have shown variable levels of chitin concentration.

## 4. Digestibility and Bioavailability of Nutrients from BSFL

BSFL have been used as a potential alternative source of animal feed, especially for poultry, due to their high digestibility and bioavailability of nutrients.

### 4.1. Protein Digestibility

The utilization of nutrients from BSFL is typically efficient. Larval composition allows for the effective utilization of nutrients by broilers, leading to improved growth performance. The protein digestibility of BSFL in poultry is a topic of interest for many researchers and producers, as BSFL have a high protein content and a low environmental impact. The protein digestibility of BSFL in animals and poultry may vary depending on the larvae meal’s age, processing method, and inclusion level. Some studies have reported that BSFL can be used as a partial or complete replacement for conventional protein sources in animal feeds, such as soybean meal, fish meal, or insect meal [40,59,60,61,62]. Recent studies on the protein digestibility of BSFL reveal that the dried larvae protein exhibits a digestibility of 48%, highlighting the need for efficient processing methods to enhance its nutritional value [63]. When the larvae biomass is defatted, the protein digestibility significantly improves to 75%, indicating the impact of fat content on protein availability [63]. The high protein content in BSFL, constituting up to 50% of their dry weight, positions them as a potential sustainable alternative to traditional protein sources in animal feeds [56]. BSFL full-fat meal has a high protein content and is a good source of amino acids, which can further improve CP digestibility [64]. Another interesting recent study has investigated the relationship between inclusion level and nutrient digestibility by using the full-fatted BSFL in diets and found that a moderate inclusion (3%) of full-fatted BSFL in the diet significantly enhanced the digestibility of crude protein [59]. This indicated that these nutrients are better absorbed and used even at lower inclusion levels. The requirement for a balanced approach in feed formulation is shown by the adverse effects of more extensive BSFL inclusions (9%) on nutritional digestibility [59].

### 4.2. Fat Bioavailability

BSFL also contain significant amounts of lipids (fats), providing a source of energy for broilers. The fat bioavailability of BSFL in poultry refers to how effectively poultry can digest and utilize the fats present in BSFL as a food source. BSFL are increasingly recognized as a sustainable and efficient protein source for animal feed, including poultry diets. The concept of fat bioavailability is crucial in animal nutrition, as it influences the energy value of the feed and the health of the animals. The lipid profile of BSFL is a significant factor in determining their bioavailability as a feed ingredient, particularly in poultry diets. This profile is characterized by a high content of saturated fatty acids (SFAs), with lauric acid (C12:0) being the most abundant [55,65]. A study demonstrated that larvae with higher weight generally contain a higher percentage of saturated fatty acids and a lower percentage of unsaturated fatty acids such as eicosapentaenoic acid (EPA) and docosahexaenoic acid (DHA) [66]. Furthermore, research on the fatty acid composition of BSFL revealed a high content of saturated fatty acids, including a significant 58.9% concentration of lauric acid [67]. Incorporating BSFL fat into poultry diets has emerged as an innovative approach with multiple benefits, as evidenced by various studies. The dietary supplementation of BSFL oil has been shown to positively influence broiler health, suggesting its efficacy as an alternative fat source [68]. This is further supported by research on the inclusion of BSFL fat in finisher broiler chicken diets, which maintains growth performance while offering a sustainable alternative to conventional fat sources [69]. Additionally, evaluating low inclusion rates of full-fatted BSFL meal in chicken diets indicates improvements in growth performance, nutrient digestibility, and gut health [59]. This underscores the potential of BSFL as a comprehensive feed ingredient, as reviewed in studies highlighting BSFL meal’s promise for poultry nutrition. The fatty acid composition of BSFL, notably influenced by the feed substrate, presents significant applications in the feed industry, offering a balanced and nutritious feed component [70]. Research also points to the possibility of BSFL meal and fat completely replacing soybean cake and oil in diets for laying hens, indicating a substantial shift in traditional poultry feeding practices [71]. The impact of BSFL larva fat on broiler diets extends to the quality of the chicken meat itself, particularly affecting breast meat quality [72]. Furthermore, modified BSF larva fat in broiler diets has been examined for its effects on performance, carcass traits, blood parameters, histomorphological features, and gut microbiota, demonstrating its comprehensive impact on poultry health and product quality [73]. Overall, the bioavailability of fat from BSFL in poultry diets presents a multifaceted opportunity. It not only aligns with sustainable feed production but also enhances the nutritional profile of poultry diets, thereby contributing to healthier poultry and potentially higher-quality poultry products.

### 4.3. Mineral Uptake

BSFL are known to be a good source of minerals and vitamins, contributing to the overall nutritional value of broiler diets. One of the main advantages of BSFL is their high calcium content, which can reach up to 9% of dry matter (DM) [40]. Calcium is essential for bone formation and eggshell quality in poultry, and its deficiency can cause rickets, osteomalacia, and poor production performance [74,75,76]. BSFL can provide sufficient calcium to meet the requirements of broilers and layers, and may even reduce the need for supplemental limestone or oyster shell in the diet [77]. Moreover, BSFL can enhance calcium absorption and retention in poultry, as they contain a natural form of calcium carbonate that is more soluble and bioavailable than the synthetic forms [51]. Another vital mineral in BSFL is phosphorus, which can range from 0.7% to 1.5% of DM [40]. Phosphorus is involved in many metabolic processes, such as energy production, nucleic acid synthesis, and acid–base balance. Phosphorus deficiency can impair growth, bone development, and egg production in poultry [78,79]. However, most of the phosphorus in plant-based feed ingredients is in the form of phytate, which is poorly digested and utilized by poultry and can also reduce the availability of other minerals, such as calcium, zinc, and iron. Therefore, poultry diets usually require supplemental inorganic phosphorus sources, such as monocalcium phosphate or dicalcium phosphate, which are costly and can have negative environmental impacts. BSFL can offer a more sustainable and efficient source of phosphorus for poultry, as they contain mainly non-phytate phosphorus that is highly digestible and bioavailable [51]. Furthermore, BSFL can improve phytate degradation and phosphorus utilization in poultry, as they contain phytase enzymes that can hydrolyze phytate and release its bound phosphorus [49]. Besides calcium and phosphorus, BSFL also contains other essential minerals, such as magnesium, potassium, sodium, iron, zinc, copper, manganese, and selenium, in varying amounts depending on the substrate and processing method [40]. These minerals play important roles in various physiological functions, such as enzyme activity, antioxidant defense, immune response, and blood formation. However, the optimal levels and ratios of these minerals in poultry diets are not well established, and their interactions with other dietary components may affect their absorption and metabolism. Therefore, more research is needed to determine the effects of BSFL on the mineral balance and status of poultry and to optimize the inclusion levels and combinations of BSFL with other feed ingredients.

### 4.4. Chitin as a Fiber Source

The digestibility of BSFL may be affected by the fibrous material called chitin. Although some research suggests that the chitin concentration may have an impact on how nutrients are utilized, overall effects appear to be beneficial. Recent studies have underscored the potential of chitin, a polysaccharide found in the exoskeleton of insects such as BSFL, as a sustainable and beneficial fiber source in poultry diets [80]. BSFL efficiently converts organic waste into valuable nutrients like proteins, lipids, and chitin, offering an environmentally friendly solution to waste management while providing nutrient-rich feed for poultry [81,82,83]. The chitin content in BSFL, which varies based on factors like rearing substrate and life stage, has been reported to range between 5.6% to 6.7% on a DM basis, as indicated in the study findings [84,85,86,87]. This variability is crucial for determining the nutritional impact on poultry. Several studies have explored chitin’s role in enhancing nutrient digestibility in poultry. They suggest that a moderate inclusion level of this insoluble fiber stimulates the development of the gizzard and the production of digestive enzymes, thereby improving the digestion of starch, lipids, and other dietary components [88,89,90]. Additionally, chitin’s impact on poultry gut health is notable. Chitin can modulate the intestinal microbiota, enhance immune responses, and strengthen the mucosal barrier, potentially increasing the population and diversity of beneficial gut bacteria [90,91,92]. This can lead to the production of short-chain fatty acids and a lower intestinal pH, thus inhibiting pathogenic bacteria growth. Moreover, the conversion of chitin into chitosan, its deacetylated form, has been shown to positively affect poultry plasma lipid profiles. Different studies have demonstrated that chitosan can reduce total plasma cholesterol and low-density lipoprotein (LDL) cholesterol concentrations, enhancing the high-density lipoprotein (HDL) to total cholesterol ratio in broilers [88,93,94,95,96]. These findings collectively suggest that chitin from BSFL could be a valuable component in poultry diets, potentially improving nutrient utilization, feed efficiency, gut health, antioxidant function, and lipid metabolism [96,97,98]. However, further systematic research is needed to fully understand the optimal conditions and mechanisms of chitin from BSFL action in poultry nutrition.

### 4.5. Factors Influencing Nutrient Utilization

Utilizing BSFL in poultry diets has garnered increasing interest due to their rich nutritional profile and sustainable production potential. The effectiveness of BSFL as a feed ingredient largely depends on their nutritional composition, which includes high levels of protein, essential amino acids, and lipids. These nutritional characteristics are influenced by the larvae’s age and developmental stage, as well as their diet, which can vary significantly based on the substrates they are fed [14,99]. The processing methods applied to BSFL, such as drying or pelleting, also play a crucial role in determining their digestibility and nutrient availability [100]. Furthermore, the inclusion rate of BSFL in poultry diets needs careful consideration, as it can impact feed intake, nutrient absorption, and overall poultry performance. Studies have shown that while moderate inclusion rates can be beneficial, higher rates might lead to reduced palatability and intake [101]. Palatability itself is a significant factor, influencing poultry’s acceptance of BSFL-based feeds. The texture and taste of the larvae can affect feed consumption patterns, which in turn impacts nutrient utilization [102]. Anti-nutritional factors present in BSFL, such as chitin, also warrant attention, as they can impede nutrient absorption and digestion [46]. The specific poultry species and age group being fed BSFL-based diets can exhibit varied responses due to differences in nutrient requirements and digestive capabilities. For instance, the dietary requirements of laying hens differ from those of broilers, which could affect how nutrients from BSFL are utilized [103]. The health status and gut health of the birds are also crucial, as they significantly influence nutrient absorption and overall feed efficiency [104]. Finally, environmental conditions like temperature and housing can affect feed intake, metabolic rates, and growth performance, thereby impacting the utilization of nutrients derived from BSFL [105,106,107,108]. Understanding these complex interactions is essential for optimizing the use of BSFL in poultry diets and ensuring the health and productivity of the birds. Ongoing research in this area is crucial to provide insights into maximizing the benefits of BSFL in sustainable poultry nutrition.

## 5. Performance Parameters in Poultry Fed with BSFL

### 5.1. Growth Performance

In every industry, achieving maximum profitability is key, which means securing the highest quality at the lowest possible cost. This is particularly true for those in agriculture and livestock farming, who are constantly seeking ways to enhance efficiency, especially when it comes to broiler chickens bred for meat consumption. These birds are a staple in our daily diets, prepared in a multitude of ways [109]. To optimize the growth of broilers and achieve the desired quality, producers and farmers typically rely on feed with soybean meal as a primary protein source. However, this practice can constrain their profit margins, as soybean meal is costly in animal feed [110]. Balancing the cost of quality feed to maximize profits remains a significant challenge in the poultry industry. BSFL meal is being explored as a promising solution to the challenge of costly poultry feed. The growth performance of broiler chickens is a critical measure to assess the effectiveness of their diets. Factors such as diet composition, consumption levels, efficient digestion, and proper nutrient absorption are all reflected in the growth performance metrics [111]. A comprehensive analysis of past research reveals varying outcomes regarding the impact of BSFL on broiler chicken growth as shown in Table 2. In conducting a comprehensive literature review, particularly studies by Dabbou et al. [21], Facey et al. [112], Fruci et al. [113], Mat et al. [114], Nampijja et al. [115], Bellezza Oddon et al. [116], Ipema et al. [117], Seyedalmoosavi et al. [118], Attia et al. [119], Heita et al. [120], and Murawska et al. [121], a detailed understanding emerges regarding the impact of both defatted (DF) and full-fat (FF) BSFL on the growth performance of different broiler chicken breeds. The earlier set of studies, focusing on DF BSFL, provides insights into how varying inclusion levels of this alternative protein source affect broiler chickens. In the research conducted by Dabbou et al. [21] on Ross 308 chickens, it was observed that at lower inclusion levels (5% to 10%), there were minimal changes in feed intake (FI), feed conversion ratio (FCR), and body weight gain (BWG). However, at the highest inclusion level of 15%, a significant reduction in BWG was noted, suggesting a possible threshold for the tolerable inclusion of DF BSFL in broiler diets. This finding is pivotal in understanding the optimal levels of BSFL inclusion for maximizing growth without adverse effects. Extending this research, Facey et al. [112] and Fruci et al. [113] examined Ross × Ross 708 chickens, varying the inclusion levels of DF BSFL up to 100%. These studies indicated that while lower levels (up to 25%) had a minimal or neutral impact on growth parameters, higher levels (50% and above) led to significant declines in BWG, pointing toward the diminishing returns of increasing BSFL inclusion in broiler diets. The study by Mat et al. [114] on Cobb-500 chickens over 42 days offered further insights. Replacing fish and soybean meal with DF BSFL, this study found that lower inclusion levels yielded positive growth outcomes, with a notable increase in BWG at 4%. Yet a contrasting decline in growth performance was evident at 8% and 12% inclusion levels, marked by reductions in FI and BWG, alongside an increase in FCR.

Turning to studies on FF BSFL, Nampijja et al. [115] explored its impact on Cobb-500 chickens over 28 days, substituting fish meal with FF BSFL. Their findings highlighted a trend of declining FI and BWG with increasing inclusion levels, culminating in a marked increase in FCR at 100% inclusion. This pattern underscores the potential negative implications of higher FF BSFL inclusion levels on broiler chicken growth performance. In a similar vein, research by Bellezza Oddon et al. [116], Ipema et al. [117], and Seyedalmoosavi et al. [118] on Ross 308 chickens showed that the impact of FF BSFL varies depending on the inclusion level. Some levels resulted in a decrease in FI and an improvement in BWG, whereas others, particularly at higher inclusion rates, suggested potential negative impacts. Furthermore, in recent studies by Attia et al. [119] and Heita et al. [120], the effects of 5% and 10% FF BSFL inclusion in the diets of Arbor Acres and Ross-308 broiler chickens were examined over 42 days. Attia et al. [119] reported that a 5% inclusion level of FF BSFL in Arbor Acres chickens did not significantly alter FI, FCR, or BWG. Conversely, Heita et al. [120] observed significant changes in Ross-308 chickens at the same 5% inclusion level, with a notable decrease in FI and a substantial increase in BWG. Increasing the inclusion to 10% continued to show a significant decrease in FI and a marked increase in BWG, suggesting a breed-specific response to FF BSFL. Extending the range of inclusion levels, Murawska et al. [121] reported that higher levels of FF BSFL (50%, 75%, and 100%) in Ross-308 chickens resulted in significant decreases in FI and dramatic reductions in BWG, with the most pronounced effects observed at the highest inclusion level.

From these comprehensive studies, it is evident that the inclusion of BSFL (both DF and FF) in broiler diets must be carefully calibrated because the impact of BSFL on growth performance has not yet been conclusively determined. Lower inclusion levels generally exhibit minimal negative impacts or even beneficial effects on growth performance. However, higher inclusion levels are consistently linked with adverse outcomes, particularly a decline in BWG. This relationship suggests a delicate balance in utilizing BSFL as an alternative protein source, where their benefits are maximized and negative impacts minimized. The variations in response across different breeds and inclusion levels also underscore the need for breed-specific diet formulations and a thorough understanding of the nutritional and physical properties of BSFL in poultry nutrition.

### 5.2. Meat Quality and Color

Recent studies have explored the effects of varying levels of BSFL inclusion on the meat quality of broilers, yielding insights into carcass characteristics, physical and chemical qualities, and the fatty acid profile of the meat (Table 3). Studies by Schiavone et al. [77], Pieterse et al. [122], Cullere et al. [20], and Aprianto et al. [123] reported negligible changes in dressing percentage across different BSFL inclusion levels, suggesting that BSFL does not significantly affect the overall yield of the carcass. However, studies by Altmann et al. [124] and Murawska et al. [121] provide a different perspective at higher inclusion levels. Altmann et al. [124] observed a significant increase in dressing percentage at a 50% BSFL inclusion level. On the other hand, Murawska et al. [121] reported significant changes at higher inclusion levels, with both 75% and 100% BSFL diets leading to marked decreases in dressing percentage and increment in abdominal fat. This suggests that BSFL could influence carcass yielding. Turning to the physical quality of broiler meat, the impact of BSFL is again varied. The series of studies conducted by Schiavone et al. [125] along with the investigations by Pieterse et al. [122] and Cullere et al. [20] indicated a consistent maintenance of pH levels irrespective of the BSFL inclusion rates. This consistency points toward a minimal effect of BSFL on this critical parameter of meat quality. This stability in pH is crucial, as it is a key determinant of meat freshness, shelf life, and consumer acceptability. Aprianto et al. [123] observed a notable trend of increased water-holding capacity (WHC) in the meat, corresponding with varying levels of BSFL inclusion. Enhanced WHC is indicative of improved meat juiciness and texture, which are vital for consumer satisfaction and can potentially increase the meat’s market value. Studies have revealed a steady pattern in terms of drip loss across various BSFL inclusion rates. This uniformity in drip loss is indicative of stable moisture release from the meat post-slaughter, a factor critical to meat preservation and quality. However, Aprianto et al. [123] reported decreases in cooking loss (CL). This decrease in CL suggests that BSFL inclusion may enhance the meat’s ability to retain moisture during cooking, potentially leading to alterations in its texture and juiciness. Expanding upon these findings, Murawska et al. [121] explored the effects of high BSFL inclusion levels (50%, 75%, and 100%) and observed significant changes in physical quality parameters. Their study reported notable modifications in pH at elevated BSFL inclusion levels. This shift in pH is significant as it influences meat quality, shelf-life, and microbial stability. Furthermore, Murawska et al. [121] found substantial decreases in cooking loss. Protein content, a key marker of meat quality and nutritional value, showed variations in the Aprianto et al. [123] study, suggesting that BSFL inclusion can impact protein levels. Fat content, crucial for flavor and energy value, also exhibited changes; particularly, Aprianto et al. [123] found a significant decreasing trend at different inclusion levels. This could imply a potential modification in the energy density and flavor profile of the meat. Ash content, indicative of mineral content and overall meat quality, was another variable affected by 100% inclusion as reported by Schiavone et al. [125]. The fatty acid profile, specifically saturated fatty acids (SFAs), monounsaturated fatty acids (MUFAs), and polyunsaturated fatty acids (PUFAs), is a critical aspect of meat quality and human health. Schiavone et al. [77] observed significant increases in MUFAs and decreases in PUFAs at all BSFL inclusion levels, indicating that BSFL can alter the fatty acid composition toward a higher MUFA content. Similarly, another study showed a significant increase in SFAs, and a decrease in PUFAs with 50% and 100% BSFL inclusion, suggesting a dose-dependent effect of BSFL on fatty acid composition. Moreover, Cullere et al. [20] also noted considerable changes in the fatty acid profile at high inclusion rates (50% and 100%). There was a marked increase in SFAs, along with a significant decrease in MUFAs and PUFAs. Altmann et al. [124] reported an increase in SFAs and MUFAs at a 50% inclusion level, further substantiating the idea that BSFL can significantly alter the fatty acid profile of broiler meat.

Meat color significantly impacts consumer preference and perceived meat quality, making it an essential aspect of end-product evaluation. The color of meat, primarily influenced by factors such as muscle composition and diet, serves as a primary indicator of freshness and quality for consumers. In an analysis of various studies on the impact of BSFL inclusion in broiler diets, a pattern emerges highlighting the complex relationship between diet and meat coloration, which is shown in Table 4. Murawska et al. [121] demonstrated that higher levels of BSFL (50–100%) in chicken diets led to redder breast meat. This aligns with the findings of Aprianto et al. [123], where even lower BSFL inclusion levels (1–3%) significantly enhanced meat redness and yellowness. Popova et al. [126] observed a significant increase in lightness and a decrease in yellowness in both breast and thigh meat at a moderate BSFL inclusion level (5%). This is in contrast to Cullere et al. [20] and Cullere et al. [127], who reported minimal changes across a range of inclusion levels, suggesting that the impact of BSFL on meat color might also depend on other dietary factors or the inherent properties of the meat. De Souza Vilela et al. [128] found minimal changes in lightness, redness, and yellowness at all inclusion levels. Kim et al. [129] noted changes at a 5% inclusion level with an increase in yellowness in breast meat but not in thigh meat. These studies collectively suggest that the impact of BSFL on meat color involves multiple factors. The changes in redness across the studies imply alterations in myoglobin concentration or muscle oxygenation [128]. The variations in lightness and yellowness might be attributed to changes in muscle fiber composition or fat content, influenced by the nutrient composition of BSFL [118]. Further research is needed to fully understand these interactions and determine the optimal BSFL inclusion levels for desired meat quality outcomes.

## 6. Health Benefits of BSFL in Poultry Nutrition

### 6.1. Immunity and Blood Biochemistry

**Figure 2:**
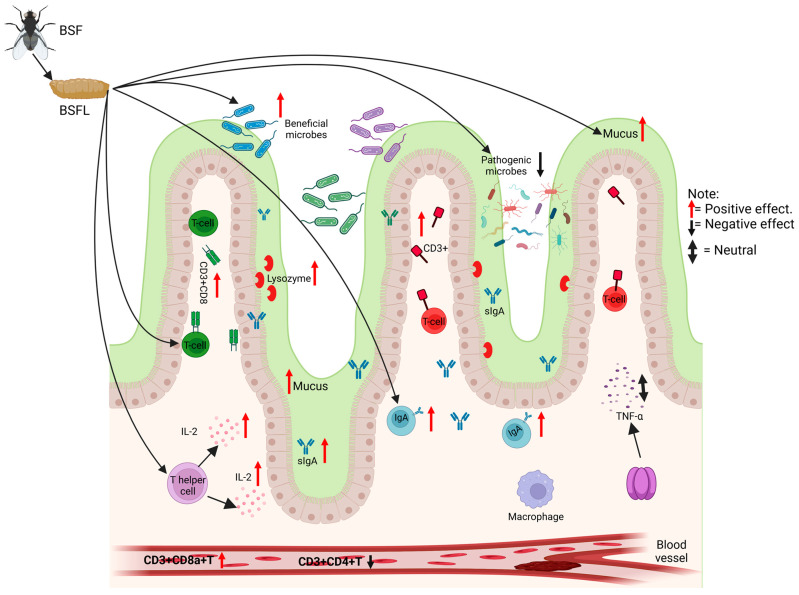
The diagram illustrates an overview of the impact of BSFL dietary supplementation on the immune response in broiler chickens. The introduction of BSFL appears to enhance the proliferation of beneficial microbes and their associated positive effects, such as increased mucus and sIgA production, as well as elevated lysozyme activity, which are essential for protective gut functions. Simultaneously, BSFL supplementation seems to modulate the immune response to pathogenic microbes. T cells, specifically CD3+ T cells, are depicted as central to this immune modulation, with T helper cells influencing the process via IL-2 secretion.

Immunity, functioning as the body’s primary defense mechanism against external pathogenic agents, is critically dependent on the integrity and functionality of the mucosal layer enveloping the gastrointestinal tract. Figure 2 illustrates the diverse effects on immune response when fed a diet based on BSFL. This mucosal barrier plays a pivotal role in preventing the entry of pathogens and maintaining homeostasis within the gut environment. This layer, rich in mucins, plays a crucial role in protecting the intestinal epithelium and maintaining overall gut health [130,131]. Dietary supplements, especially BSFL, have been found to influence this protective mucus layer in broiler chickens. Biasato et al. [132] discovered that a modest 5% inclusion of BSFL meal enhances mucin levels in the intestinal villi, potentially bolstering the mucus barrier and positively influencing the gut microbiota. However, this study also noted that higher BSFL levels (10% and 15%) led to a decrease in mucin staining intensity, suggesting a potential reduction in mucus production or increased degradation. Despite these alterations, there were no significant impacts on other hematochemical or histological factors. Further exploring the immunological effects of BSFL, Chen et al. [133] highlighted its positive impact on intestinal immunity. They observed that increased levels of BSFL significantly boosted key immune markers such as Immunoglobulin A (IgA) and Interleukin 2 (IL-2), in addition to enhancing antioxidant enzyme activities, while not notably affecting Tumor Necrosis Factor-alpha (TNF-a) levels. This indicates a potential for BSFL to strengthen immune responses in broilers. Contrastingly, de Souza Vilela et al. [128] reported that higher BSFL inclusions (up to 20%) in broiler diets resulted in substantial changes in the immune system. Notable reductions in peripheral white blood cells and lymphocytes were observed, including a 35.9% decrease in white blood cells and a 50% reduction in lymphocytes at the highest BSFL levels. Furthermore, there was a significant decline in intraepithelial CD3+T lymphocytes, with a notable decrease in both CD3+T lymphocytes and CD3+CD8 cytotoxic T lymphocytes. On the other hand, the ingestion of BSF by broilers did not have any noticeable impact on the populations of intestinal T helper (CD3+CD4+) intraepithelial lymphocytes. In the same context, Tykałowski et al. [134] observed distinct changes in T cell populations correlating with both the age of the chickens and the level of BSFL inclusion in their diet. Specifically, chickens fed with a 75% BSFL diet exhibited an elevated proportion of CD3+CD4+ T cells in their blood samples. In contrast, those on a 100% BSFL diet showed a notable reduction in these cells, coupled with an increase in CD3+CD8a+T cells, particularly at 21 days of age. Additionally, the study revealed that an increased BSFL ratio in the diet led to a higher percentage of CD3+CD8a+T cells and a lower percentage of CD3+CD4+T cells in both blood and spleen samples in chickens aged 42 days. In another related study, Lee et al. [22] found that BSFL in broiler diets increased CD4+T lymphocyte frequency, enhanced serum lysozyme activity, and improved spleen lymphocyte proliferation. A dose-dependent increase in CD3+CD4+T lymphocytes was particularly evident in BSFL-fed groups. This increase in serum lysozyme activity suggests an enhanced ability of phagocytes to destroy pathogens. Moreover, the study also documented an improved survival rate against *Salmonella gallinarum* in broilers, particularly with a 3% BSFL diet, demonstrating the capability of BSFL to elicit non-specific immune responses and enhance pathogen clearance. These studies collectively underscore the complex and dose-dependent impact of BSFL on the immune system of broiler chickens. While BSFL can augment specific immune responses and enhance pathogen defense mechanisms, carefully considering inclusion levels is necessary to avoid potential adverse effects on other aspects of the immune system.

The investigation into blood parameters offers valuable insights into the health status of broilers. The influence of BSFL on various blood parameters reflects the complex interplay between dietary components and the physiological processes in poultry. Aprianto et al. [123] and Kim et al. [129] both focus on the changes in lipid profiles, with different BSF supplementation levels affecting HDL cholesterol and total cholesterol. These alterations could stem from the unique fatty acid composition in BSFL products, directly influencing lipid metabolism. This is complemented by the observations of El-Kaiaty et al. [135], who noted increases in protein and albumin levels with BSF supplementation, indicative of enhanced liver function or improved nutritional status. Li et al. [136] also found that replacing soybean oil with BSFL fat did not negatively impact blood traits in broilers, highlighting the nutritional adequacy of BSFL-based diets. Furthermore, Seyedalmoosavi et al. [118] reported increases in uric acid and alkaline phosphatase with higher BSF levels, suggesting possible metabolic adjustments in response to the altered nutrient profile in the diet. Contrastingly, Dabbou et al. [21] and Loponte et al. [137] did not observe significant changes in serum iron and magnesium levels in similar dietary conditions. Bongiorno et al. [138] documented a reduction in gamma-glutamyl transferase activity and changes in white blood cell counts, hinting at the immunomodulatory effects of BSF. This aligns with the lack of impact on AST and ALT activity reported by Dabbou et al. [21] and others, indicating that BSF may not have a detrimental effect on liver health. Dabbou et al. [21] specifically noted that replacing soybean meal with BSFL meal did not significantly alter most blood serum parameters, except for an increase in phosphorus concentration, particularly at a 10% dietary inclusion of BSF larvae meal. This contrasts with the findings of Neumann et al. [139] and Schiavone et al. [69], who observed changes in growth performance and fatty acid composition in broiler meat when BSF meal was used. Collectively, these studies suggest that BSFL can induce a range of biochemical changes in broilers, influenced by factors such as the level of supplementation and the specific form of BSFL used. These changes encompass aspects of lipid metabolism, liver function, immune response, and overall nutritional status, highlighting the diverse impact of BSF in poultry nutrition. Further investigation is essential to comprehend these effects fully and to optimize the use of BSFL in poultry diets for enhanced health and performance.

### 6.2. Gastrointestinal Health and Microbiota

Incorporating BSFL in broiler diets has been shown to significantly impact gastrointestinal health, as evidenced by changes in intestinal morphology across various studies. Chen et al. [133] demonstrated that in broilers, BSFL oil supplementation led to notable variations in the gut. In the duodenum, there were no significant differences in villus height and crypt depth between the control and treatment groups. In contrast, the jejunum showed an increase in villus height, suggesting a differentiated impact of BSFL along the intestinal tract. The ileum responded uniquely, with distinct measurements in villus height and crypt depth, underscoring the complex influence of BSFL on intestinal morphology. Similarly, Kim et al. [68] found that replacing soybean with 50% BSFL resulted in increased villus height in the ileum, enhancing the absorptive surface area crucial for gut health. However, this study noted no significant differences in crypt depth, the villus height to crypt depth ratio, villus width, and villus area among treatments. In another perspective, Dabbou et al. [21] reported that higher inclusion levels of BSFL meal in broiler diets led to shorter villi and shallower crypts in the gut, along with a reduced villus height-to-crypt depth ratio. These morphological changes suggest potential alterations in digestive and absorptive functions in the intestinal tract. These findings highlight the multifaceted effects of BSFL on broiler gastrointestinal health, pointing toward their significant role in modifying gut morphology and function.

According to several studies, the inclusion of BSFL in chicken diets has been linked to both beneficial and harmful impacts on the gut microbiota. Ndotono et al. [140] found that a diet with 75% BSFL significantly increased beneficial lactic acid bacteria, enhancing gut microbiota diversity. However, Biasato et al. [132] noted that while a 5% inclusion of BSFL meal fostered beneficial genera, a 15% inclusion led to reduced microbial complexity and an increase in mucolytic bacteria, potentially harming gut health. De Souza Vilela et al. [86] observed that up to 20% BSFL in diets reduced the abundance of certain bacteria like Enterococcus and unclassified *Christensenellaceae*, while positively correlating with beneficial butyrate-producing bacteria such as *Roseburia* and *Dehalobacterium*. In contrast, Ndotono [141] reported an increase in potentially pathogenic bacteria like Campylobacter and *Clostridia* at higher BSFL meal inclusion rates, indicating a risk associated with higher concentrations. An analogous finding was reported by another research study, which found that incorporating BSFL at a level of 50% lowered specific constituents of the microbial community residing in the cecum, including potentially pathogenic bacteria like *Enterobacteriaceae* and the *Bacteroides-Prevotella* group. Commensal populations, including *Bacillus* spp., *C. leptum* subgroup, and *C. coccoides*-*Eubacterium rectale* group, were also restricted [142]. In a study conducted by Attia et al. [119], the influence of different protein sources, including BSFL, on cecal microbial counts in broiler chickens was examined, yielding significant results on the effects of BSFL. The study found no substantial differences in the counts of clostridial, total coliform, and *Escherichia coli* among the groups fed with fish meal (FM), BSFL, and black soldier fly pupae (BSFP) at both 3% and 5% inclusion levels during the starter and growing–finishing phases. However, a distinct variation was noted in groups fed with BSFP, which exhibited markedly lower Salmonella counts and increased *Lactobacillus* spp. counts in the cecum compared with the control group. Moreover, these groups demonstrated higher overall bacterial counts than all other groups, except those exclusively fed with BSFL. The collective results from these studies emphasize the critical necessity of finely tuning the inclusion rates of BSFL in poultry diets. This careful adjustment is key to harnessing the benefits for gut health and reducing any potential negative impacts. There is a clear need for more comprehensive and systematic research to further unravel the complex interactions between the gut microbiota and BSFL in the diets of broiler chickens, which could provide deeper insights into optimizing poultry nutrition.

## 7. Economic Analysis of Using BSFL in Poultry Feeding

Recent studies have highlighted the economic benefits of incorporating BSFL into poultry diets, revealing their potential to significantly reduce feed costs while maintaining or enhancing production efficiency. Waithaka et al. [143] observed that diets with 20% BSFL yielded the highest cost–benefit ratio (CBR) of 2.12, suggesting that BSFL can effectively lower feed costs and make meat and egg products more affordable. In a similar vein, another study reported positive gains in profitability and return on investment (RoI) with increased levels of BSFL substitution for fish meal, indicating its affordability and sustainability as an alternative feed source [144]. Additionally, in a study by Mutisya et al. [145], broiler chickens fed diets with higher proportions of BSFL showed greater economic returns compared with conventional diets, underscoring the financial viability of BSFL in poultry nutrition. Moreover, a recent study identified an optimal substitution level of 540 g/kg of BSFL meal that resulted in a 23% decrease in feed cost per bird without compromising growth or meat quality, although the benefits diminished with higher substitution levels [115]. These studies collectively demonstrate the potential of BSFL to enhance the economic efficiency of poultry farming, offering a sustainable and cost-effective alternative to traditional protein sources in poultry feed.

## 8. Future Directions in Research and Development

With the increasing global need for sustainable and affordable poultry nutrition options, the role of BSFL as an alternative protein source is attracting more attention. Nonetheless, numerous research areas are either not yet explored or have been insufficiently investigated, offering the potential for future research endeavors.

### 8.1. Nutritional Optimization

The role of BSFL as a promising alternative protein source for poultry feed is increasingly recognized, especially given their high protein and fat content. However, the nutritional value of BSFL is subject to variation based on the type and quality of the organic waste they feed on. Therefore, tailoring their diet during the larval stage is pivotal for maximizing their potential as poultry feed ingredients. Research has delved into how various feeding substrates affect the amino acid profile, fatty acid composition, and micronutrient levels of BSFL. Zulkifli et al. [49] conducted a comparative study on the nutritional value of BSFL meals processed via spray-drying and oven-drying, revealing that spray-dried BSFL exhibited superior protein, amino acid, and nucleotide contents compared with their oven-dried counterparts. According to Cammack et al. [146], the optimal diet for BSFL should be high in protein and carbohydrates, with a 70% water content. Furthermore, Nyakeri et al. [147] developed an effective feeding strategy for enhancing BSFL biomass production and reducing fecal sludge, finding that BSFL nourished with a blend of fecal sludge and vegetable waste yielded higher crude protein and lower crude fat levels than those fed exclusively on fecal sludge. A recent study has found that BSFL fed on animal feed substrates has the highest levels of polyunsaturated fatty acids [148]. In contrast, another study discovered that a combination of fermented fruit wastes and tofu by-products led to BSFL with high crude protein levels but lower crude fat, ash, calcium, and phosphorus [149]. Additionally, a different study revealed that the chemical composition of BSFL is influenced by the substrate’s particle size and fiber content [150]. These findings suggest that the type and physical attributes of the substrate significantly affect the nutritional profile of BSFL.

Despite these variations, the Association of American Feed Control Officials (AAFCO) advocates for the use of locally sourced, pre-consumer food waste as a substrate for BSFL rearing [151]. This recommendation is based on the premise that such waste contains valuable nutrients that would otherwise be discarded in landfills, composts, or converted to energy, thus providing a sustainable and nutrient-rich feeding option for BSFL cultivation. Thus, investigating diverse feeding substrates for BSFL and their resultant impact on the larvae’s nutritional profile is crucial for uncovering insights that could significantly improve their application in poultry nutrition.

### 8.2. Impact on Poultry Health and Product Quality

Feed additives and unconventional feed ingredients play crucial roles in poultry production to improve nutrition, enhance growth, and health, as well as optimize product quality [152,153,154,155,156,157,158,159,160]. The effects of BSFL-based diets on poultry health, including immune function, disease resistance, and overall physiological well-being, are not fully understood. Similarly, the influence of BSFL on the quality of poultry products, such as meat, warrants deeper exploration to assess parameters like taste, texture, nutritional value, and shelf life. Several studies have examined the impact of BSFL on these aspects and reported mixed results [77,78,79,80,81,82,83,84,85,86,87,88,89,90,91,92,93,94,95,96,97,98,99,100,101,102,103,104,105,106,107,108,109,110,111,112,113,114,115,116,117,118,119,120,121,122,123,124,125,126,127,128]. Therefore, more research is needed to optimize the inclusion level, processing method, and origin of the BSFL, as well as the poultry species, breed, and age, to ensure the safety and quality of poultry products.

### 8.3. Sustainable Production and Processing Methods

Enhancing the sustainability and efficiency of BSFL as a feed source necessitates advancements in production and processing techniques. The growth, survival, and nutrient composition of BSFL are influenced by various factors, including rearing conditions, processing methods, and the approach to converting waste into feed. Optimizing these factors is crucial for increasing both the yield and quality of BSFL. Research by Purkayastha and Sarkar [161] identified the ideal temperature and humidity for BSFL rearing as 30 °C and 70%, respectively. Additionally, Ee et al. [7] discovered that maintaining a ratio of 100 g of BSFL per kg of substrate maximizes biomass production and waste reduction. Peguero et al. [162] have shown that spray-drying and microwave-drying are more energy-efficient and less environmentally harmful than traditional oven-drying and sun-drying methods for processing BSFL. Furthermore, Ee et al. [7] indicated that pretreatment of maize straw with alkali and steam explosion enhances its digestibility and bioconversion efficiency in BSFL. In a similar vein, research by Phi et al. [163] demonstrated that co-digesting food waste and sewage sludge with BSFL not only reduces pathogen levels but also enriches the nutrient profile of the larvae.

### 8.4. Environmental Impact Assessment

BSFL have been proposed as a sustainable alternative to traditional feed sources like soybean meal and fishmeal, which contribute to deforestation, overfishing, and greenhouse gas emissions. Comprehensive life cycle assessments (LCAs) are essential to evaluate the actual environmental benefits of BSFL. Studies, such as the one by Smetana et al. [164], have shown that BSFL meal, when used as protein in rainbow trout feed, results in lower greenhouse gas emissions and land and water use compared with soybean and fishmeal, although it has a higher energy use and eutrophication potential due to electricity consumption and nutrient leaching in BSFL production. This impact can be mitigated by using organic waste as a substrate, which reduces emissions from waste disposal and fertilizer production. Similarly, Weththasinghe et al. [165] found that BSFL oil is a sustainable alternative to fishmeal and fish oil in aquaculture. These findings suggest that BSFL can reduce reliance on traditional feed sources, potentially lowering environmental impacts like greenhouse gas emissions. However, the findings also highlight challenges in BSFL rearing, such as ammonia emissions and nutrient leaching, contributing to environmental issues like acidification and eutrophication. These impacts could be reduced by using renewable energy and improving nutrient management in BSFL systems. Additionally, further research and LCA are needed for broiler production to fully understand the environmental impacts and benefits of incorporating BSFL, thus representing a significant area for future research.

### 8.5. Consumer Acceptance and Regulatory Considerations

The market success of BSFL as a novel feed ingredient hinges significantly on consumer acceptance and regulatory approval. Research into consumer attitudes toward poultry products derived from BSFL-based diets reveals varying acceptance levels, influenced by factors such as consumer knowledge, perceptions, and cultural backgrounds. A recent study by Khaemba et al. [166] indicated that about 70% of consumers are open to purchasing poultry products fed on BSFL-based diets. Similarly, Harriet et al. [167] discovered that most consumers informed about insect meals showed interest in buying meat from insect-fed poultry. Furthermore, the regulatory landscape for BSFL varies internationally and is shaped by regional legal frameworks and safety standards. In the United States, the Food and Drug Administration (FDA) has suggested revising the Association of American Feed Control Officials (AAFCO) ingredient definition for dried BSFL to include its use in poultry feed. Meanwhile, in the European Union, the European Commission has authorized the use of BSFL as feed for poultry and pigs. Therefore, effectively addressing both consumer and regulatory challenges is key to the development and wider adoption of BSFL as a sustainable feed source. Therefore, navigating regulatory landscapes and obtaining approvals for BSFL use in various regions will be a key area of focus.

### 8.6. Long-Term Health Studies

The long-term health effects of BSFL-based diets on poultry are poorly understood. Several studies have evaluated the short-term effects of BSFL on the growth performance, immune function, and product quality of poultry, and reported positive or neutral results [113,118,123,133,140]. However, these studies were mainly conducted for a few weeks or months and did not consider the cumulative effects of BSFL across different life stages and generations of poultry. Therefore, conducting long-term studies to assess the impact of BSFL on the health and welfare of poultry, as well as the potential risks of disease transmission, bioaccumulation, and allergenicity, is essential to ensure the safety and sustainability of this novel feed source [143].

### 8.7. Optimizing BSFL Use in Feed Formulation and Processing for Economic Viability

To optimize BSFL use, in-depth research should be conducted to examine the economic feasibility of incorporating BSFL as an insect as such or meal into poultry feed. This involves streamlining BSFL production to reduce costs, refining feed formulation to maximize nutritional benefits and feed efficiency, and improving processing techniques to ensure pellet quality and stability [40,168]. Furthermore, research should focus on developing cost-effective methods for integrating BSFL into various types of poultry diets, analyzing the cost–benefit ratio compared with conventional feeds, and identifying market opportunities for BSFL-based products. Studies should also explore innovative processing technologies to optimize the use of BSFL in feed while maintaining their nutritional integrity.

## 9. Conclusions

Expanding on utilizing BSFL in broiler diets, the research in this domain is increasingly encouraging. It is crucial to remain updated with the latest advancements to enhance various aspects of poultry farming, including production efficiency, dietary inclusion level, and meat quality. The sustainable nature of BSFL, grown on organic waste, aligns with environmentally friendly and circular agricultural practices. Their substantial protein content, constituting 32% to 53% of their DM, positions BSFL as a significant source of amino acids, which are essential for the growth and health of broiler chickens. Current studies consistently demonstrate that incorporating BSFL into broiler chicken diets can yield growth performance that is on par with or better than diets based on conventional protein sources, underscoring their effectiveness as an alternative feed option.

## Figures and Tables

**Table 1:** Chemical composition of BSFL.

Type	CP (%)	CF (%)	Crude Fiber (%)	Ash (%)	Chitin (%)	References
FF	43.1	38.6	4.1	2.7	6.7	[55]
FF	41.1	30.1	0.0	9.3	0.0	[56]
FF	43.9	29.4	21.3	13.2	0.0	[53]
FF	35.0	29.8	7.9	5.3	0.0	[57]
FF	40.1	32.5	0.0	10.4	0.0	[58]
FF	27.54	51.53	0.0	6.59	3.87	[54]
DF	55.42	9.85	7.4	8.1	7.21	[54]
DF	65.5	4.6	0.0	9.3	6.9	[51]
DF	21.6	6.3	7.0	9.3	0.0	[50]
SM	49.44	1.4	7.43	7.19	0.0	[52]
FM	67.53	10.36	0.26	17.15	0.0	[52]

CP: crude protein; CF: crude fat; FF: full fat; DF: defatted; SM: soybean meal; FM: fish meal.

**Table 2:** Effects of inclusion of various levels of BSFL on the growth performance in broiler chicken.

Birds	Duration (Days)	Type of BSFL	Substitute to	Inclusion (%)	FI (%)	FCR (%)	BWG (gm)	References
Ross 308	35	DF	SM	5	NS (−1.02)	NS (−0.6)	NS (−5.14)	[21]
Ross 308	35	DF	SM	10	NS (−1.22)	NS (0.0)	NS (9.7)	[21]
Ross 308	35	DF	SM	15	NS (−2.66)	*** (−7.5)	*** (−197.1)	[21]
Ross × Ross 708	49	DF	SM	12.5	NS (−0.39)	NS (−2)	NS (45)	[112]
Ross × Ross 708	49	DF	SM	25	NS (0.12)	NS (−1.83)	NS (71)	[112]
Ross × Ross 708	49	DF	SM	50	NS (−4.28)	*** (3.95)	*** (−248)	[112]
Ross × Ross 708	49	DF	SM	100	*** (−10.05)	*** (9.39)	*** (−547)	[112]
Ross × Ross 708	35	DF	SM	12.5	NS (−1.77)	NS (−1.55)	NS (7.20)	[113]
Ross × Ross 708	35	DF	SM	25	NS (−0.98)	NS (0.91)	NS (−29.90)	[113]
Ross × Ross 708	35	DF	SM	50	NS (−2.12)	NS (4.93)	** (−97.70)	[113]
Ross × Ross 708	35	DF	SM	100	** (−18.52)	** (9.57)	** (−286.20)	[113]
Cobb-500	42	DF	FM and SBM	4	** (6.08)	** (5.6)	*** (152.3)	[114]
Cobb-500	42	DF	FM and SM	8	** (−10.33)	** (44.4)	*** (−178.5)	[114]
Cobb-500	42	DF	FM and SM	12	** (−34.04)	** (−5.6)	*** (−204.7)	[114]
Cobb-500	28	FF	FM	25	NS (−8.99)	NS (1.99)	NS (−122.21)	[115]
Cobb-500	28	FF	FM	50	* (−15.78)	NS (−5.47)	NS (−123.04)	[115]
Cobb-500	28	FF	FM	75	* (−20.13)	NS (3.48)	*** (−258.96)	[115]
Cobb-500	28	FF	FM	100	* (−37.39)	*** (21.89)	*** (−544.97)	[115]
Ross 308	38	FF		5	NS (−1.82)	NS (0.73)	NS (39)	[116]
Ross 308	35	FF		8	*** (−6.96)	*** (−0.8)	*** (98)	[117]
Ross-308	42	FF		10	NS (−0.17)	NS (5.03)	NS (−81)	[118]
Ross-308	42	FF		20	NS (8.46)	NS (6.29)	NS (−45)	[118]
Ross-308	42	FF		30	NS (4.83)	*** (17.61)	NS (−137)	[118]
Arbor Acres	42	FF	SM	5	NS (13.39)	NS (9.84)	NS (−11)	[119]
Ross-308	42	FF	SM	5	* (−30.42)	NS (25.64)	* (364.6)	[120]
Ross-308	42	FF	SM	10	* (−23.90)	NS (6.15)	* (178.48)	[120]
Ross-308	42	FF	SM	50	** (−10.12)	NS (−2.64)	** (−319)	[121]
Ross-308	42	FF	SM	75	** (−10.53)	NS (−2.27)	** (−541.5)	[121]
Ross-308	42	FF	SM	100	** (19.92)	* (8.53)	** (−668)	[121]

Numbers in parentheses indicate the percentage of change compared with the control group. FI: feed intake; FCR: feed conversion ratio; BWG: body weight gain; DF: defatted; FF: full fat; SM: soybean meal; FM; fish meal; NS: not significant; *: *p* < 0.05; **: *p* < 0.01; ***: *p* < 0.001.

**Table 3:** Effects of different inclusion levels of BSFL on the meat quality of broiler chicken.

Inclusion (%)	Bird Number	Dressing (%)	Abdominal Fat (%)	Physical Quality				Chemical Quality			Fatty Acid Profile			References
				pH (%)	WHC (%)	DL (%)	CL (%)	Protein (%)	Fat (%)	Ash (%)	SFA (%)	MUFA (%)	PUFA (%)	
5	64	NS (0.31)	* (−0.04)	NS (−0.04)	n.a	NS (0.12)	NS (0.38)	n.a	n.a	n.a	NS (−0.55)	*** (8.66)	*** (−5.43)	[77]
10	64	NS (0.37)	* (0.22)	NS (0.01)	n.a	NS (0.02)	NS (−0.8)	n.a	n.a	n.a	NS (0.38)	*** (14.34)	*** (−10.55)	[77]
15	64	NS (0.45)	* (0.23)	NS (−0.05)	n.a	NS (0.15)	NS (−0.4)	n.a	n.a	n.a	NS (2.57)	*** (16.52)	*** (−13.48)	[77]
1	70	NS (−0.23)	* (−0.17)	n.a	** (5.79)	n.a	* (−0.65)	*** (0.07)	*** (−0.9)	NS (−0.22)	*** (3.31)	NS (0.14)	NS (−2.07)	[123]
2	70	NS (−0.23)	* (−0.27)	n.a	** (6.89)	n.a	* (−0.75)	*** (0.52)	*** (−1.73)	NS (−0.41)	*** (1.97)	** (0.88)	** (−5)	[123]
3	70	NS (0.04)	* (−0.36)	n.a	** (10.13)	n.a	* (−2.53)	*** (1.26)	*** (−1.15)	NS (−0.25)	*** (2.83)	** (1.13)	** (−4.38)	[123]
5	80	NS (0.3)	n.a	NS (−0.63)	n.a	NS (0.16)	NS (−1.47)	NS (−0.1)	NS (0)	NS (0)	n.a	n.a	n.a	[122]
10	80	NS (0.4)	n.a	NS (−0.94)	n.a	NS (1.06)	NS (−4.24)	NS (−0.3)	NS (−0.1)	NS (0)	n.a	n.a	n.a	[122]
15	80	NS (−0.8)	n.a	NS (−0.94)	n.a	NS (0.48)	NS (0.76)	NS (0.1)	NS (0.3)	NS (0)	n.a	n.a	n.a	[122]
50	49	* (9.2)	n.a	n.a	n.a	NS (0.02)	NS (2.71)	n.a	n.a	n.a	* (18.15)	* (12.96)	* (−13.64)	[124]
50	40	NS (0.54)	n.a	NS (1.28)	n.a	NS (−0.01)	n.a	NS (0.3)	NS (−0.01)	NS (0.05)	**** (26.74)	*** (−7.58)	**** (−16.85)	[20]
100	40	NS (3.53)	n.a	NS (0.96)	n.a	NS (−0.02)	n.a	NS (−0.1)	NS (0.28)	NS (0.1)	**** (59.03)	*** (−15.16)	**** (−36.41)	[20]
50	50	NS (3.84)	NS (−0.2)	NS (0.52)	n.a	NS (1.2)	n.a	NS (0)	NS (0.06)	NS (0)	**** (17.39)	NS (1.32)	**** (−15.49)	[125]
100	50	NS (0.75)	NS (0.2)	NS (1.37)	n.a	NS (0.4)	n.a	NS (0)	NS (−0.14)	* (−0.01)	**** (35.09)	NS (0.88)	**** (−28.26)	[125]
50	96	*** (−1.3)	*** (0.34)	*** (3.10)	n.a	NS (−0.2)	*** (−2.96)	n.a	n.a		n.a	n.a	n.a	[121]
75	96	*** (−6)	*** (0.92)	*** (−3.26)	n.a	NS (−0.01)	*** (−4.73)	n.a	n.a		n.a	n.a	n.a	[121]
100	96	*** (−3.7)	*** (1.15)	*** (3.10)	n.a	NS (−0.18)	*** (−4.64)	n.a	n.a		n.a	n.a	n.a	[121]

Numbers in parentheses indicate the percentage of change compared with the control group. WHC: water-holding capacity; DL: drip loss; CL: cooking loss; SFA: saturated fatty acid; MUFA: monounsaturated fatty acid; PUFA: polyunsaturated fatty acid; n.a: not analyzed; NS: not significant; *: *p* < 0.05; **: *p* < 0.01; ***: *p* < 0.001; ****: *p* < 0.0001.

**Table 4:** Effects of various inclusion levels of BSFL on meat color.

Inclusion (%)	Birds Nr	Type of Meat	L*	a*	b*	References
50	96	Breast	** (−6.21)	** (12.44)	*** (−20.63)	[121]
75	96	Breast	** (−5.49)	** (33.61)	*** (−6.30)	[121]
100	96	Breast	** (−4.93)	** (20.17)	*** (−12.75)	[121]
1	70	Breast	NS (−2.41)	*** (14.95)	** (8.54)	[123]
2	70	Breast	NS (−2.56)	*** (75.25)	** (27.07)	[123]
3	70	Breast	NS (−4.56)	*** (53.61)	** (35.87)	[123]
5	50	Breast	*** (9.53)	NS (−13.63)	* (−21.11)	[126]
50	40	Breast	NS (−0.64)	NS (17.65)	NS (10.85)	[20]
100	40	Breast	NS (2.12)	NS (5.43)	NS (12.40)	[20]
10	150	Breast	NS (0.55)	* (39.51)	NS (5.03)	[127]
15	150	Breast	NS (−0.73)	* (−43.21)	NS (1.29)	[127]
2.5	80	Breast	NS (2.06)	NS (−2.38)	NS (59.5)	[128]
5	80	Breast	NS (0.86)	NS (−3.89)	NS (78.6)	[128]
7.5	80	Breast	NS (1.37)	NS (−2.59)	NS (71.4)	[128]
10	80	Breast	NS (2.06)	NS (−9.07)	NS (114.3)	[128]
5	150	Breast	NS (−1.82)	NS (32.19)	** (15.88)	[129]
5	150	Thigh	NS (1.16)	NS (−7.23)	NS (5.71)	[129]
5	50	Thigh	**** (9.43)	NS (−18.47)	* (−24.47)	[126]

Numbers in parentheses indicate the percentage of change compared with the control group. Nr: number; L*: lightness; a*: redness; b*: yellowness; NS: not significant; *: *p* < 0.05; **: *p* < 0.01; ***: *p* < 0.001; ****: *p* < 0.0001.

## Data Availability

The supporting data of this study are available within the article.
